# Amplitude differences in high-frequency fMRI signals between eyes open and eyes closed resting states

**DOI:** 10.3389/fnhum.2014.00503

**Published:** 2014-07-08

**Authors:** Bin-Ke Yuan, Jue Wang, Yu-Feng Zang, Dong-Qiang Liu

**Affiliations:** ^1^Hangzhou Institute of Service Engineering, Hangzhou Normal UniversityHangzhou, China; ^2^Center for Cognition and Brain Disorders, Hangzhou Normal UniversityHangzhou, China; ^3^Zhejiang Key Laboratory for Research in Assessment of Cognitive Impairments, Hangzhou Normal UniversityHangzhou, China

**Keywords:** resting state fMRI, high-frequency fluctuations, fluctuation amplitude, eyes open, eyes closed

## Abstract

Recent studies employing rapid sampling techniques have demonstrated that the resting state fMRI (rs-fMRI) signal exhibits synchronized activities at frequencies much higher than the conventional frequency range (<0.1 Hz). However, little work has investigated the changes in the high-frequency fluctuations between different resting states. Here, we acquired rs-fMRI data at a high sampling rate (TR = 400 ms) from subjects with both eyes open (EO) and eyes closed (EC), and compared the amplitude of fluctuation (AF) between EO and EC for both the low- and high-frequency components. In addition to robust AF differences in the conventional low frequency band (<0.1 Hz) in visual cortex, primary auditory cortex and primary sensorimotor cortex (PSMC), we also detected high-frequency (primarily in 0.1–0.35 Hz) differences. The high-frequency results without covariates regression exhibited noisy patterns. For the data with nuisance covariates regression, we found a significant and reproducible reduction in high-frequency AF between EO and EC in the bilateral PSMC and the supplementary motor area (SMA), and an increase in high-frequency AF in the left middle occipital gyrus (MOG). Furthermore, we investigated the effect of sampling rate by down-sampling the data to effective TR = 2 s. Briefly, by using the rapid sampling rate, we were able to detect more regions with significant differences while identifying fewer artifactual differences in the high-frequency bands as compared to the down-sampled dataset. We concluded that (1) high-frequency fluctuations of rs-fMRI signals can be modulated by different resting states and thus may be of physiological importance; and (2) the regression of covariates and the use of fast sampling rates are superior for revealing high-frequency differences in rs-fMRI signals.

## Introduction

Resting state fMRI (rs-fMRI) has widely been used to investigate spontaneous activity of human brain. Most rs-fMRI studies have focused on signal fluctuations at frequencies of <0.1 Hz (Biswal et al., 1995; Lowe et al., 1998; Cordes et al., 2000, 2001; Greicius et al., 2003; Fox and Raichle, 2007); in contrast, high-frequency (>0.1 Hz) components are generally regarded as physiological noises and are therefore discarded. Recently, however, the rs-fMRI high-frequency signal has attracted increased attention. By decomposing the full bandwidth into four frequency bands (0.01–0.05, 0.05–0.1, 0.1–0.15 and 0.15–0.2 Hz), Baria et al. (2011) found that the signal power of limbic and paralimbic regions is mainly located in high-frequency bands (0.1–0.2 Hz), and that the visual ventral stream exhibited a graded shift of power from low- to high-frequency bands, suggesting that a closed relationship exists between the anatomical structures and the full spectrum profiles of brain oscillations. Furthermore, high-frequency (>0.1 Hz) specific abnormalities have been detected in some disease states, including chronic somatic pain (Malinen et al., 2010; Baliki et al., 2011; Otti et al., 2013) and visceral pain (Hong et al., 2013), implying that the high-frequency information present in the rs-fMRI signal could provide useful information in clinical studies.

Moreover, using rapid-sampling-rate rs-fMRI, a few groups have observed some temporally coherent networks in frequency bands far above the conventional range (Boubela et al., 2013; Lee et al., 2013). For example, using a high-temporal-resolution MR-encephalography (MREG) sequence (TR = 100 ms), Lee et al. (2013) observed visual and sensorimotor networks in both low- and high-frequency bands. Using a multi-band EPI sequence with a TR of 354 ms, default mode network (DMN) and fronto-parietal network were also identified at 0.25–1.4 Hz (Boubela et al., 2013). Despite the great promise of high sampling rates for revealing the fast oscillations of rs-fMRI, it is yet not known whether such high-frequency fluctuations can be modulated by different resting states.

The aim of the present study was twofold. First, using a high sampling rate of 400 ms, we sought to determine whether the fast fluctuations of the rs-fMRI signal could be modulated by different resting states. In particular, we examined changes in the fluctuation amplitude of high-frequency rs-fMRI signals between eyes open (EO) with no fixation and eyes closed (EC) resting states in healthy adults. EEG studies have revealed that the EO-EC differences occur in almost all frequency bands of the EEG power spectrum (Barry et al., 2007; Chen et al., 2008). Previous rs-fMRI studies have also demonstrated that EO-EC differences in the amplitude of low frequency fluctuation (ALFF; Zang et al., 2007; Mcavoy et al., 2008) were highly reproducible across subjects (Yang et al., 2007; Liu et al., 2013), suggesting the changes in brain oscillations between continuous states of EO and EC are very robust. However, to date, few studies examined high-frequency BOLD signal changes between continuous states of EO and EC. Here, we extended the amplitude calculation to the full bandwidth and examined the changes in the high-frequency fluctuation amplitude.

Second, this study aimed to determine whether sampling rate affects the high-frequency results. All recent clinical studies examining high-frequency fluctuations of rs-fMRI signals have used typical temporal sampling rates (Malinen et al., 2010; Baliki et al., 2011; Hong et al., 2013; Otti et al., 2013). However, according to the Nyquist sampling theorem, if the sampling rate is not sufficiently high, the high-frequency spectrum will be aliased into lower frequencies leading to inaccurate results (Oppenheim et al., 1997). High sampling rates have been utilized in previous rs-fMRI studies to reduce such aliasing effects caused by high-frequency physiological noises (Cordes et al., 2001; Yang et al., 2007), and it has been demonstrated that the use of high sampling rate can improve sensitivity for detecting changes in ALFF between EO and EC (Yang et al., 2007). Here, we predicted that the sampling rate also affects high-frequency rs-fMRI results.

## Materials and methods

### Participants

This study was approved by the ethics committee of the Center for Cognition and Brain Disorders, Hangzhou Normal University. Forty-six healthy adults (24.8 ± 1.7 years, range 22–32 years; 23 females) participated in the study. Each participant provided written informed consent. They were screened with a questionnaire to ensure no history of brain injury, neurological illness or psychiatric disorders.

### Data acquisition

**Figure 1:**
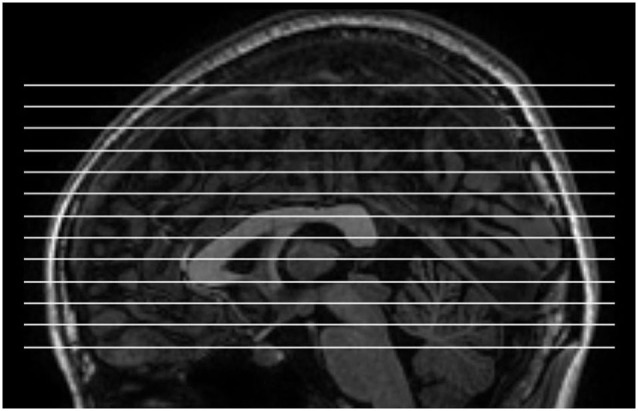
**The slice position for short-TR rs-fMRI data acquisition**. TR = 400 ms, total number of slices = 13, thickness/gap = 6 mm/1 mm.

MRI images were acquired using a GE Discovery MR-750 3.0 T scanner (GE Medical Systems, Waukesha, WI) at the Center for Cognition and Brain Disorders of Hangzhou Normal University. The subjects lay supine with the head snugly fixed by straps and foam pads to minimize movement. An rs-fMRI dataset obtained using a conventional sampling rate (TR = 2 s) was first collected. This dataset was acquired for other purposes and not analyzed here. For the purpose of spatial normalization, we acquired a 3D T1-weighted image for each subject using a spoiled gradient-recalled pulse sequence (176 sagittal slices, thickness = 1 mm, TR = 8100 ms, TE = 3.1 ms, flip angle = 8°, and FOV = 250 × 250 mm^2^). Then, short-TR EO and EC rs-fMRI data were collected. The scanning parameters were as follows: TR = 400 ms, TE = 15 ms, flip angle = 30°, thickness/gap = 6/1 mm, FOV = 240 × 240 mm^2^, and matrix = 64 × 64. The parameter set used for the short-TR fMRI data acquisition was adopted from a previous study (Yang et al., 2007), except that TE was set to 15 ms in order to obtain the maximum number of slices possible. Consequently, 13 axial slices were scanned for each subject, covering most parts of the cerebrum (Figure 1). Each of the two sessions lasted for 8 min and consisted of 1200 volumes. The order of the EO and EC sessions was counterbalanced across subjects. Additionally, the multi-slice T1 images scanned at the same slice positions as those used for the shorter-TR rs-fMRI data were obtained, using a T1-weighted fluid-attenuated inversion recovery (FLAIR) pulse sequence (13 axial slices, thickness/gap = 6/1 mm, TR = 2382 ms, TE = 25 ms, flip angle = 90°, FOV = 240 × 240 mm^2^, and matrix = 512 × 512). During resting state scanning, the subjects were instructed to keep as motionless as possible, to remain relaxed, not to think of particular things and not to fall asleep. The scanner room was kept dim during scanning. At the end of the scanning sessions, the subjects confirmed that they had not fallen asleep.

### Data preprocessing

The data were preprocessed according to the following steps:

1. For each subject, the 3D T1 image was firstly co-registered with the multi-slice T1 image, and then normalized to Montreal Neurological Institute (MNI) space. Consequently, a transformation matrix from the original space of the multi-slice T1 image to the MNI space was obtained. This step was performed by using Statistical Parameter Mapping (SPM8^1^) software package.
2. For the functional images, the first 50 volumes (20 s) of the data were discarded to avoid transient signal changes before the magnetization reached a steady-state and to allow the subjects to become accustomed to the fMRI scanning environment. After removing these volumes, each 4D dataset contained 1150 volumes.
3. Slice timing and head motion (HM) correction were performed to functional images using SPM8. No subject had HM more than 2.0 mm maximum displacement in any direction of *x*, *y*, and *z* or 2° of any angular motion throughout the course of scan.
4. Then the functional data were spatially re-sampled to 3 × 3 × 3 mm^3^. This step was performed by using the image reslicing function of REST software^2^ (Song et al., 2011).
5. Because the multi-slice T1 images were acquired immediately after the short-TR fMRI scans, we assumed that the functional images and the multi-slice T1 images were in the same space. This assumption was confirmed by careful examination of the raw data for each subject. Therefore, we did not coregister the short-TR functional images with the multi-slices T1 images. The transformation matrix (obtained in step 1) was directly applied to the spatially resampled functional images using SPM8.
6. Finally, we removed the temporal linear trend from the functional data using the REST software.

### Frequency domain analysis

In the current study, we were interested in the high-frequency fluctuations of rs-fMRI signals. Therefore, we extended the calculation of ALFF (Zang et al., 2007) towards the high-frequency bands. First, the times series were transformed to the frequency domain using fast Fourier transform (FFT), resulting in the power spectrum. Then the square root of the power spectrum was averaged across the frequency bands of interest (a low-frequency interval, a high-frequency interval and 23 sub-bands within the high-frequency interval, as described later) for each voxel. This averaged square root was taken as the amplitude of fluctuation (AF) value.

No consensus exists regarding the boundary between the low- and high-frequency ranges in the current rs-fMRI literature. In this work, we considered the frequency bands between 0.01 and 0.1 Hz as low–frequency band. Frequency range higher than 0.1 Hz was considered as high-frequency band. The upper boundary for the high-frequency range was determined by 0.5/TR = 1.25 Hz. For standardization, the AF value of each voxel was divided by the mean AF within a brain mask (Zang et al., 2007; Yan et al., 2013b). This mask was obtained from the intersection of the non-zero voxels of all subjects’ normalized functional images and a whole brain mask in REST software. Prior to the statistical analysis, the whole-brain-mean scaled AF maps were smoothed using a 6-mm full width half maximum (FWHM) Gaussian kernel. The AF calculation was performed using the REST software, and the spatial smoothing was performed using SPM8.

### Statistical analysis

Paired *t*-tests were performed for each frequency band of interest. A contiguity threshold of 37 contiguous voxels (determined by Monte Carlo simulations (Ledberg et al., 1998)) and voxel-level *p* < 0.01 were used as criteria for significant difference corresponding to a corrected *p* < 0.05 within the intersection mask. These steps were performed using the REST software. Because these analyses were exploratory in nature, we did not perform the multiple comparison correction among the frequency bands of interest.

### Nuisance covariates regression

Previous work has demonstrated that the high-frequency bands of rs-fMRI signals contain physiological noises (Cordes et al., 2001). To reduce the influence of physiological noises on the rs-fMRI signal, we repeated the AF and statistical analyses to the data with nuisance covariates regression. Because we did not simultaneously record the physiological signals (e.g., electrocardiographic and respiratory signals) during the fMRI experiment, we extracted the averaged time series from several regions of interest (ROIs) as the estimates of these physiological components. These covariable signals included (1) the averaged signal within the white matter (WM) ROI provided by the REST software; (2) the averaged cerebrospinal flow (CSF) signal within the ventricle ROI provided by the REST software; (3) the averaged signal within a spherical ROI in the suprasellar cistern (SC) (MNI coordinate: *x* = −6, *y* = −2, *z* = −17; radius = 3 mm) adjacent to the Circle of Wilis; and (4) head motion (HM) parameters. Here, we used the Friston 24-parameter HM model, which includes 6 HM parameters, 6 HM parameters one time point before and the 12 corresponding square items (Friston et al., 1996), since the Friston 24-paramter model was highly recommended because of its efficiency in HM covariate regression for rs-fMRI data (Yan et al., 2013a). The nuisance covariates regression was performed on the detrended fMRI data by using the DPARSF software (Yan and Zang, 2010). The analyses after regression were the same as those aforementioned.

Although contentious (Fox et al., 2009; Murphy et al., 2009; Weissenbacher et al., 2009; Van Dijk et al., 2010; Saad et al., 2012), the global signal is sometimes considered as a nuisance effect (Macey et al., 2004). Thus, global signal is often removed from the data by using a linear regression technique. In this study, we examined how the high-frequency AF differences were affected when adding the global signal as an extra covariable in addition to the other covariates (i.e., the WM, CSF, SC signals and HM parameters). To achieve this goal, we repeated the above analyses but added the global signal as an extra covariate in the nuisance covariate regression step. The statistical results obtained with and without global signal regression (GSR) were compared by visual inspection.

### Reproducibility assessment

To validate the findings, we assessed the split-half reproducibility of the high-frequency results. We randomly divided the subjects into two subgroups (subgroups 1 and 2), which were matched for age, gender and session order (EO and EC). We then performed conjunction analyses using SPM8. As we were interested in whether some regions showed significant differences for both subgroups, we tested against the conjunction null hypothesis (Nichols et al., 2005) that one or two subgroups did not exhibit significant differences. A contiguity threshold of 170 contiguous voxels (determined by Monte Carlo simulations (Ledberg et al., 1998)) and voxel-level *p* < 0.05 were used as criteria for significant difference corresponding to a corrected *p* < 0.05. In the conjunction map, the significance of one voxel indicates that the results are jointly significant for both subgroups (Friston et al., 2005; Nichols et al., 2005).

### Temporal down-sampling

Based on the Nyquist sampling theorem, we also investigated the effect of sampling rate on the high-frequency (>0.1 Hz) AF differences between EO and EC. As such, a given time series was down-sampled to a low temporal resolution and the results were compared to those of the original time series. Because a TR of 2 s is typical in fMRI studies, we down-sampled the preprocessed rs-fMRI time series to an effective TR of 2 s (denoted as Eff-TR = 2 s) as was done in previous work (Yang et al., 2007). This sampling rate corresponds to a frequency band of 0–0.25 Hz. Consequently, we obtained 5 down-sampled (denoted as Eff-TR2000) datasets, each containing 230 volumes, and performed the AF calculation for each set and frequency band (0.01–0.1, 0.1–0.15, 0.15–0.2 and 0.2–0.25 Hz). Then, the individual frequency-specific AF maps were averaged across the 5 sets for each condition (i.e., EO and EC). Statistical comparisons were performed on the resulting averaged AF maps for each frequency band. These analyses were also performed to the data with (WM, CSF, SC, and HM) and without covariates regression.

## Results

### EO-EC differences in the low-frequency band

**Figure 2:**
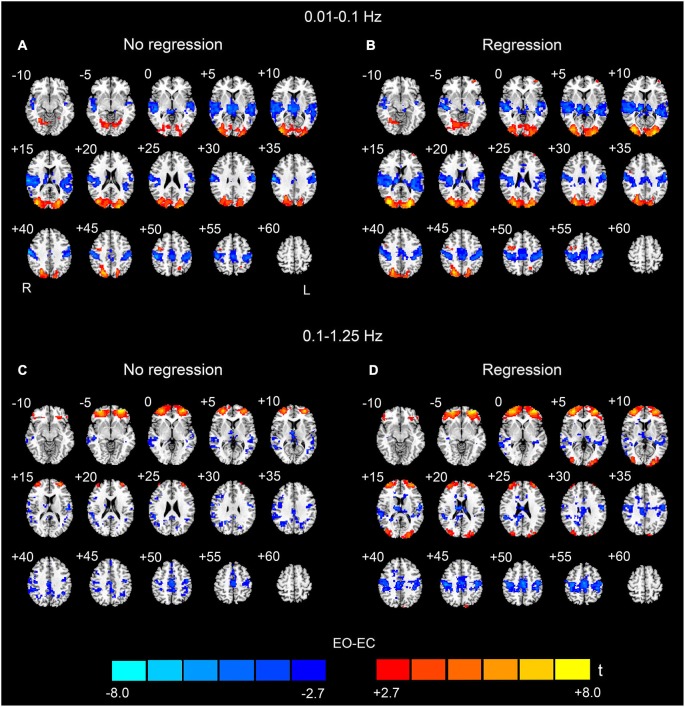
**Paired *t*-test results of the AF between EO and EC**. The upper rows (**A** and **B**) show the results for the low-frequency band (0.01–0.1 Hz), and the bottom rows (**C** and **D**) show the results for the high-frequency band (0.1–1.25 Hz) (*p* < 0.05, corrected). **(A)** and **(C)** show the EO-EC AF differences without nuisance covariates regression (No regression), and **(B)** and **(D)** show the EO-EC AF differences with nuisance covariates regression (Regression). Warm colors indicate higher AF in EO than EC, and cold colors indicate the opposite. The left side of the figure corresponds to the right side of the brain.

For the low-frequency band (0.01–0.1 Hz), we detected significantly higher AF in bilateral MOG and prefrontal regions in the EO condition than in the EC condition. We also detected lower AF in bilateral superior temporal cortex, PSMC, SMA, paracentral lobule, middle cingulum cortex and thalamus (*p* < 0.05, corrected, Figures 2A,B). The spatial patterns of the data analyzed with and without nuisance covariates regression were similar and resembled previous findings (Yang et al., 2007; Yan et al., 2009; Liu et al., 2013).

### EO-EC differences in the high-frequency band

In this study, we also detected significant EO**-**EC differences in high-frequency bands (>0.1 Hz). For the data without nuisance covariates regression, significantly increased AF in EO was observed in prefrontal cortex, however, the spatial extent of the prefrontal regions was much larger than that found for the low-frequency results. We did not find that AF was higher in the visual cortex during EO as compared to EC. The spatial extent of the regions exhibiting decreased AF was smaller than that in the low-frequency results. These regions were located in bilateral middle and superior temporal cortex, bilateral PSMC, SMA, middle cingulum cortex, inferior parietal cortex, precuneus, right angular gyrus and thalamus (*p* < 0.05, corrected, Figure 2C).

**Figure 3:**
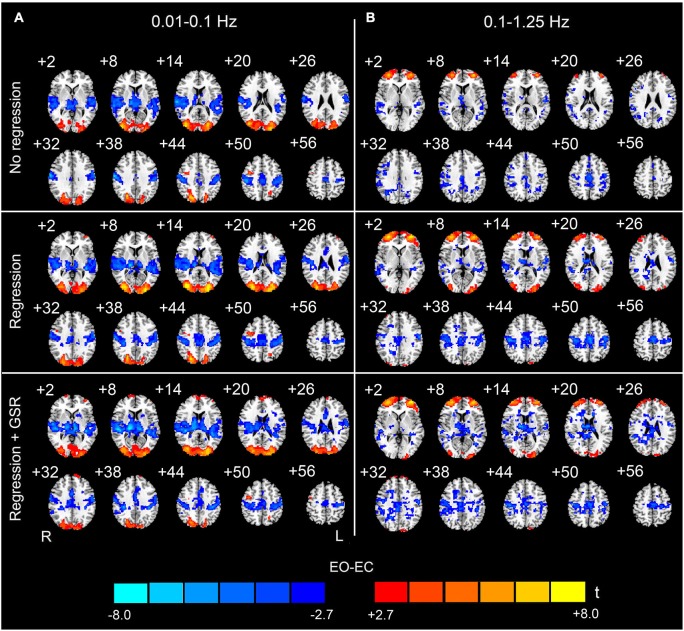
**The effect of global signal regression on the EO-EC AF differences**. The upper, middle and bottom rows show the results without any covariates regression (No regression), regression without global signal but with other covariates (WM, CSF, SC, HM) (Regression), and regression with global signal and the other covariates (WM, CSF, SC, HM) (Regression + GSR), respectively. The left column **(A)** shows the results for the low-frequency band (0.01–0.1 Hz), and the right column **(B)** shows the results for the high-frequency band (0.1–1.25 Hz) (*p* < 0.05, corrected). For the high-frequency results, the pattern with GSR was noisier than that without GSR but with other covariates regression. For the low-frequency results, the influence of GSR was not evident. Warm colors indicate higher AF in EO than EC, and cold colors indicate the opposite. The left side of the figure corresponds to the right side of the brain.

For the data with nuisance covariates regression, we detected significantly decreased AF in bilateral PSMC, SMA, primary auditory cortex, middle frontal gyrus, and thalamus. The significantly increased AF were found in bilateral MOG and anterior regions of prefrontal cortex (*p* < 0.05, corrected, Figure 2D). The results with GSR showed a similar pattern to those results without GSR, except that the results with GSR demonstrated more distributed and noisier pattern than those without GSR at high frequencies (0.1–1.25 Hz) (Figure 3).

**Figure 4:**
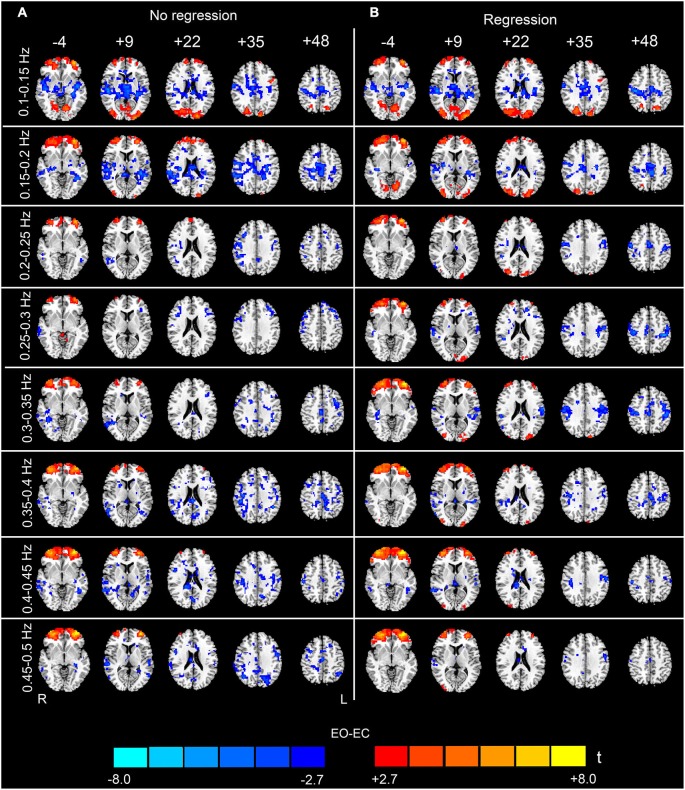
**Paired *t*-test results for the high-frequency sub-bands from 0.1 to 0.5 Hz (see the sub-bands from 0.5 to 0.9 Hz in Figure 5 and the sub-bands from 0.9 to 1.25 Hz in Figure 6)**. The left-hand column **(A)** shows the results without performing nuisance covariates regression (No regression), and the right-hand column **(B)** shows the results with nuisance covariates regression (Regression) (*p* < 0.05, corrected). The pattern of the results with covariates regression appears much cleaner than the results without covariates regression. Warm colors indicate higher sub-band-specific AF in EO than EC, and cold colors indicate the opposite. The left side of the figure corresponds to the right side of the brain.

**Figure 5:**
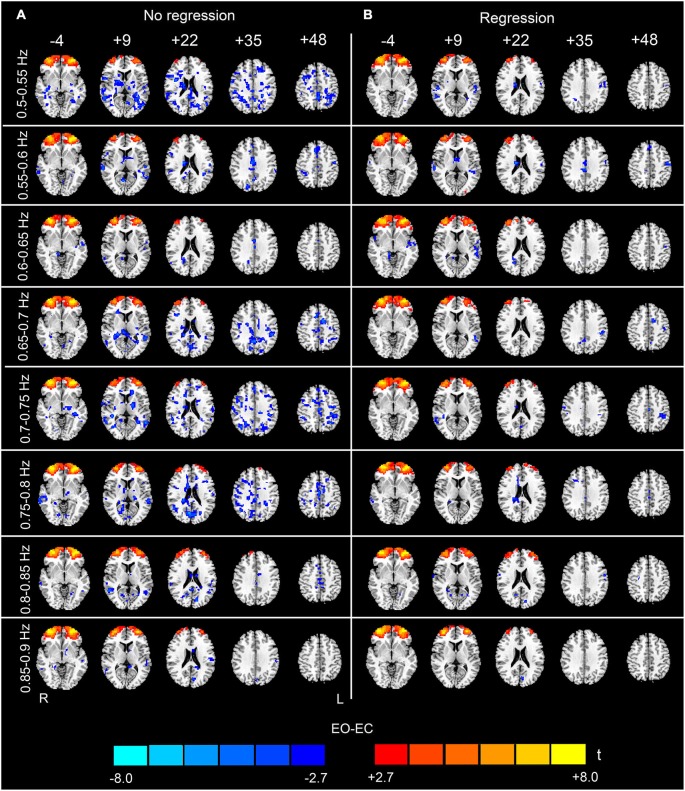
**(Continuation of Figure 4) Paired *t*-test results for the high-frequency sub-bands from 0.5 to 0.9 Hz**. For details, see Figure 4.

**Figure 6:**
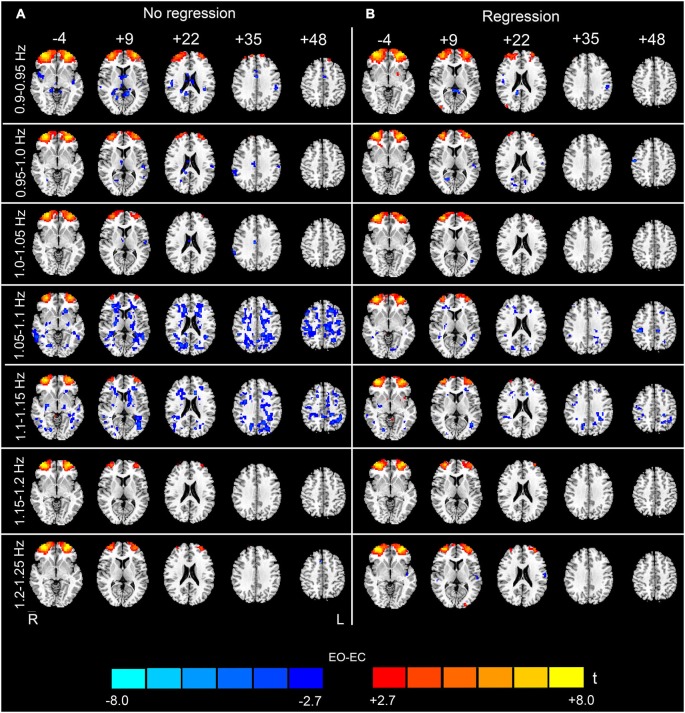
**(Continuation of Figure 5) Paired *t*-test results for the high-frequency sub-bands from 0.9 to 1.25 Hz**. For details, see Figure 4.

The analysis of the 23 high-frequency sub-bands revealed significant AF differences between EO and EC. For the data without nuisance covariates regression, significant differences were detected in most of these sub-bands. The patterns of significant differences found in the 0.1–0.2 Hz sub-bands were similar to those found in the low-frequency band, except that differences in prefrontal regions were detected. For higher frequency sub-bands, the spatial distribution of the differences was dispersed into smaller foci (*p* < 0.05, corrected, Figures 4A, 5A and 6A). For the data with nuisance covariates regression, the robust and significantly decreased AF were detected in bilateral PSMC and SMA from 0.1 to 0.45 Hz. The significant increased AF was also found in bilateral MOG in these sub-bands (*p* < 0.05, corrected, Figure 4B). The increases in AF in anterior regions of prefrontal cortex were found for all sub-bands, irrespective of nuisance covariates regression.

### Conjunction analysis results

**Figure 7:**
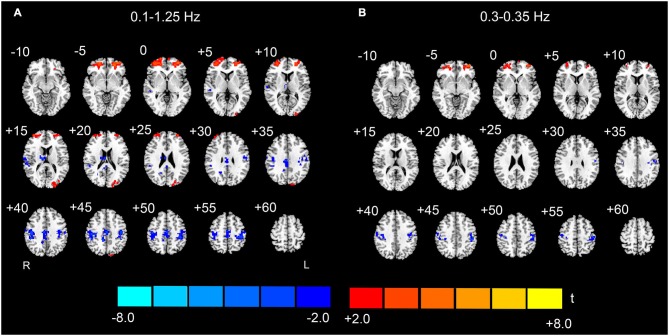
**Conjunction maps of the EO-EC high-frequency AF differences across the split-half subgroups**. The results for the entire high-frequency band (0.1–1.25 Hz) are shown on the left **(A)**, and the results of one high-frequency sub-band (0.3–0.35 Hz) are shown on the right **(B)**. Conjunction results at frequencies >0.35 Hz appear noisy and are not shown. Warm colors indicate the jointly significant increase of high-frequency AF in EO than EC, and the cold colors indicate the opposite (*p* < 0.05, corrected). The left side of the figure corresponds to the right side of the brain.

To validate the observed high-frequency results, we split the sample into two matched subgroups, and performed the conjunction analyses to reveal whether the results were reproducible across the two subgroups. Because the results with the nuisance covariates regression were less noisy (i.e., there were less abrupt changes among the spatial maps of neighboring sub-bands, and fewer distributed and false positive clusters in the WM) than those without nuisance covariates regression (Figures 4–6), the conjunction analyses were performed only on the data with covariates regression. For the entire high-frequency band (0.1–1.25 Hz), the jointly significant decreased AF was detected in bilateral PSMC and SMA. The jointly significant increased AF was found in left MOG and bilateral anterior regions of prefrontal cortex (Figure 7A). Conjunction analyses of the sub-band specific AF maps revealed consistent increases and decreases in AF between the two subgroups from 0.1 to 0.35 Hz, but the conjunction maps became noisier in higher frequency bands. For example, we still observed jointly significant increases in AF in anterior prefrontal cortex and decreases in AF in bilateral PSMC for sub-band as high as 0.3–0.35 Hz (Figure 7B). However, the conjunction maps were noisier in the high-frequency sub-bands.

### The effects of sampling rate

**Figure 8:**
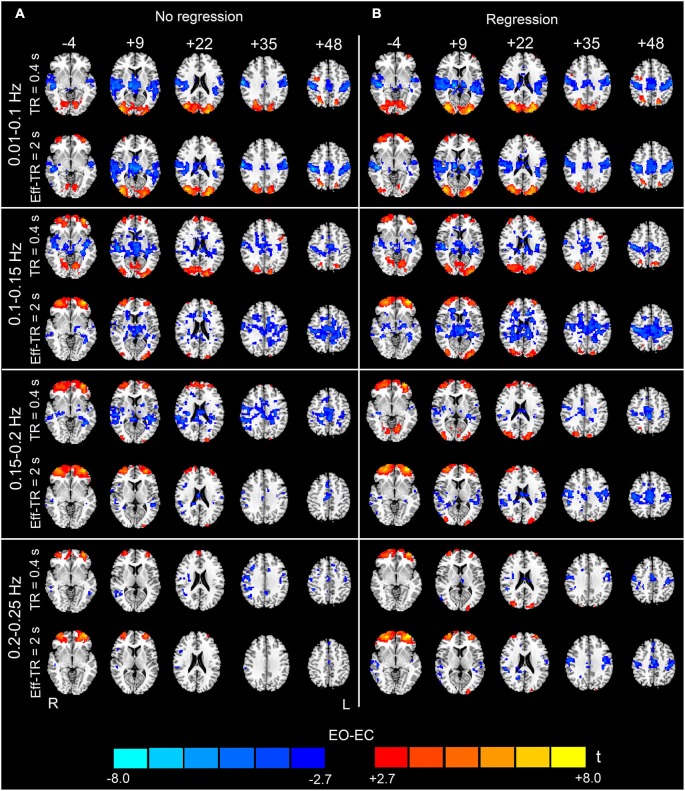
**Paired *t*-test results for the original (TR400) dataset and down-sampled (Eff-TR2000) dataset**. The results for different frequency sub-bands (0.01–0.1, 0.1–0.15, 0.15–0.2 and 0.2–0.25 Hz) are shown from the top to the bottom of the figure. The left-hand column **(A)** shows the results obtained without performing nuisance covariates regression, and the right-hand column **(B)** shows the results with nuisance covariates regression. The upper row in each panel shows the results for the TR400 dataset, and the lower row in each panel shows the results for the Eff-TR2000 dataset (*p* < 0.05, corrected). In the sub-bands between 0.15–0.25 Hz, fewer significant regions were detected in the Eff-TR2000 dataset compared to the TR400 dataset only if the covariates were not regressed out. For the 0.1–0.15 Hz sub-band, more artifactual differences in WM were detected in the Eff-TR2000 dataset, irrespective of whether covariates were regressed out. Warm colors indicate higher AF in EO than EC, and cold colors indicate the opposite. The left side of the figure corresponds to the right side of the brain.

Figure 8 shows the EO-EC differences for the original dataset (denoted as the TR400 dataset) and the Eff-TR2000 datasets, respectively. For the FFT, the maximal observable frequency is 1/(2 × TR). Therefore, for the Eff-TR2000 dataset, we obtained only the frequency spectrum in the range 0–0.25 Hz. For this reason, when comparing the results based on the Eff-TR2000 and the TR400 datasets, we only examined the frequency band of 0.01–0.25 Hz. Ultra-low frequency (<0.01 Hz) components were not a focus of this study. For the data without nuisance covariates regression, the differences found in the low frequency band (0.01–0.1 Hz) were similar between the TR400 and Eff-TR2000 datasets. However, the patterns were quite different between the two datasets when examining the high-frequency sub-bands. In the 0.1–0.15 Hz, after resampling, widely distributed differences were observed (lower AF in EO than EC) in the WM, and the extent of supra-threshold regions in the occipital lobe was smaller than that in the TR400 dataset. In 0.15–0.25 Hz, the spatial extent of supra-threshold regions was much smaller in the Eff-TR2000 dataset than in the TR400 dataset (Figure 8A).

For the data with nuisance covariates regression, differences were apparent between the TR400 and Eff-TR2000 datasets only when studying sub-bands in the range 0.1–0.15 Hz. The brain regions that exhibited significant AF for the Eff-TR2000 dataset was larger than that for the TR400 dataset. Nevertheless, some of these regions were found in the WM and were merged with the clusters in bilateral PSMC and SMA (Figure 8B). Because the data with GSR exhibited a much nosier pattern at high-frequencies (Figure 3), down-sampling was performed on the data only without GSR (i.e., the other covariates but no global signal were regressed).

## Discussion

In this study, we investigated whether the high-frequency components of rs-fMRI signals can be modulated between EO and EC resting states. We used a high sampling rate (TR = 400 ms) to acquire the rs-fMRI data, and calculated the AF of the rs-fMRI signals. We detected significant and reproducible differences in the high-frequency AF. Our findings suggest that the high-frequency rs-fMRI signal can be modulated between different resting states. Additionally, we showed that nuisance covariates regression and the temporal sampling rate affected the detection of the high-frequency AF changes.

### Changes in high-frequency fluctuations

In the present study, the AF values of both the low- and high-frequency intervals of rs-fMRI signal were calculated. Currently, no definition of the range corresponding to “low frequency” has been agreed upon. One of the most common intervals used in rs-fMRI studies is 0.01–0.1 Hz; therefore, we considered 0.01–0.1 Hz as the low-frequency interval. Frequencies higher than 0.1 Hz were considered as “high-frequency”. The low-frequency differences were located primarily in the visual cortex, auditory cortex, sensorimotor cortex and thalamus, consistent with previous findings (Yang et al., 2007; Yan et al., 2009; Liu et al., 2013). Such highly consistent results suggest that the changes in ALFF between EO and EC are robust across different scanning parameters. Importantly, we also detected significant differences in high-frequency (0.1–1.25 Hz) AF between EO and EC. The results were less noisy (i.e., there were less abrupt changes among the spatial maps of neighboring sub-bands, and fewer distributed and false positive clusters in the WM) if nuisance covariates were regressed out. By dividing the high-frequency band (0.1–1.25 Hz) into 23 sub-bands, we identified significant differences from 0.1 to 0.35 Hz.

We believe that the observed high-frequency differences in AF between EO and EC are credible for three reasons. First, although it has been demonstrated that the high-frequency rs-fMRI BOLD signal contains physiological noises (Cordes et al., 2001), the high-frequency differences in our data still remained significant even after the signals extracted from WM, ventricle and SC ROIs were regressed out from the data (Figures 4–6). Second, the statistical maps were less noisy, for the data with nuisance covariates regression, and less abrupt changes were observed in spatial patterns among neighboring sub-bands for these data than those without covariates regression (Figures 4–6). We speculated that such abrupt changes in the spatial patterns may be due to the influence of high-frequency physiological noises. Third, the conjunction analyses both for the entire high-frequency band (i.e., 0.1–1.25 Hz) and for the individual sub-bands (e.g., 0.3–0.35 Hz) revealed that the two subgroups, with no overlapping subjects, had jointly significant AF changes in regions such as the bilateral PSMC, suggesting reproducible high-frequency differences across people (Friston et al., 2005; Nichols et al., 2005).

Therefore, our results suggest that the observed AF differences in the high-frequency range are not due to physiological noises but might have functional significance. Our results are in line with several recent studies that used high-sampling techniques to examine the functional connectivity of high-frequency rs-fMRI signals. Either by seed based correlation (Lee et al., 2013) or independent component analysis (ICA; Boubela et al., 2013), it was found a few networks including motor network, visual network, fronto-parietal network and DMN demonstrated synchronized activities at frequency bands much higher than those studied conventionally. Although we utilized a different measure, the AF, both our findings and previous functional connectivity findings suggest that the high-frequency bands of rs-fMRI signals contain meaningful information and should not be ignored. More importantly, our results suggested that the fast fluctuations of rs-fMRI signals can be modulated by different resting states. The EO-EC differences can be found at the frequency bands much higher than the conventional “low frequency band”. Currently, the mechanisms that underlie the high-frequency EO-EC differences are not fully understood. A recent study reported that the effects caused by volitional changes in EO and EC were independent of changes in the exogenous visual stimuli (Jao et al., 2013). We speculated the decrease in high-frequency fluctuations in primary auditory cortex and PSMC during EO might be involved in the cross modal inhibition process (Laurienti et al., 2002), i.e., the ongoing activities of both auditory cortex and PSMC are suppressed when subjects keep their EO. Further investigation is needed to improve our understanding of the functional and behavioral significance of such neuroimaging findings.

Although the current experiment was performed on healthy subjects, the observed high-frequency AF differences in some regions, such as the motor network, suggest that it would be interesting to examine the high-frequency**-**specific abnormalities in patients with motor disorders, such as Parkinson’s disease (PD) because it has been shown that the motor network showed altered spontaneous brain activity in PD patients (Wu et al., 2009, 2011). In addition, resting brain activity exhibits some frequency-specific features in PD patients (Esposito et al., 2013). As more clinical studies seek to identify the frequency specificity of the rs-fMRI signals (Malinen et al., 2010; Baliki et al., 2011; Baria et al., 2011; Wee et al., 2012; Esposito et al., 2013; Yu et al., 2014), studies of high-frequency information within the rs-fMRI signals can add to our understanding of the frequency spectrum profiles of the spontaneous brain activity in healthy population (Baria et al., 2011) and in patients with brain disorders (Wee et al., 2012). Such studies might provide novel biomarkers for use in the investigation of the mechanisms underlying some neurological disorders. Thus, the functional significance of the high-frequency component of rs-fMRI fluctuations requires further investigation.

A prominent finding in this study is the existence of a robust increase in high-frequency AF in bilateral anterior regions of the prefrontal cortex. Differences such as this were much more apparent in the high-frequency (>0.1 Hz) than in the low frequency (0.01–0.1 Hz) bands (Figure 2) and were found to be significant in every sub-band within the frequency range 0.1–1.25 Hz; this findings was almost independent of the uses of covariates regression (Figures 4–6). These results indicate that such differences are specifically found at high frequencies. However, because the locations exhibiting such differences were closed to the eye balls, it is unclear whether such differences were caused by brain activity or by artifacts resulting from eye movement. Therefore, it is difficult to interpret the significance of these differences. Unfortunately, the current study did not record eye movements. In future investigations, simultaneous fMRI and electro-oculography (EOG) measurements (Yoon et al., 2005) would be helpful to improve our understanding of the mechanisms underlying this phenomenon. Another methodological limitation in this work is that we verified that the short-TR fMRI images and the multi-slice T1 images matched based on visual inspection only. Subtle differences between their positions might exist, which would increase error in the results. More quantitative alignment approaches could be used to correct for any such bias.

Previous work has demonstrated that the differences in ALFF between EO (without fixation) and EC are quite reproducible across subjects (Yang et al., 2007; Liu et al., 2013), suggesting that the underlying changes in spontaneous brain activity between the two types of resting state are very stable. In this study, to obtain more credible conclusions, we used the EO-EC paradigm to examine the high-frequency changes of rs-fMRI signals. However, significant differences in both ALFF (Yan et al., 2009) and functional connectivity measures (Yan et al., 2009; Patriat et al., 2013) have been shown between EO with fixation and without fixation states; therefore, it would be interesting to examine frequency changes among these three resting states.

### The effects of nuisance covariates regression

In this study, we found that nuisance covariates regression greatly influenced the detection of high-frequency AF differences. Our results demonstrated that the overall patterns of data without nuisance covariates regression appeared noisy in most sub-bands, e.g., in sub-bands of 0.65–0.8 Hz and in 1.05–1.15 Hz (see Figures 5, 6). Abrupt changes were also observed in the spatial patterns among neighboring sub-bands (e.g., 0.3–0.6 Hz and 0.8–0.95 Hz, see Figures 4–6). In contrast, the patterns with the nuisance covariates regression were much cleaner. Some brain regions (e.g., MOG and PMSC) exhibited significant high-frequency AF differences between EO and EC, which was consistent with the low frequency differences. Based on our results, we conclude that the effect of physiological noises should be considered in analysis of high-frequency rs-fMRI signals.

We also examined the effect of GSR on the detection of high-frequency AF differences between EO and EC. Although GSR has a large impact on functional connectivity results (Fox et al., 2009; Murphy et al., 2009; Weissenbacher et al., 2009; Van Dijk et al., 2010; Saad et al., 2012), our results showed that it had little impact on the traditional ALFF differences between EO and EC. For the high-frequency results, GSR yielded to a noiser pattern. Our results also support the assumption that global signal may not be a very good estimate of the global nuisance effect (Murphy et al., 2009; Chai et al., 2012).

### The effects of sampling rate

Additionally, our data showed that the sampling rate had non-negligible effects on the EO-EC high-frequency AF differences. According to the Nyquist sampling theorem, for a given band-limited time series, if the sampling rate is insufficient to capture the high-frequency information present, then the high-frequency components will be aliased into the lower-frequency bands of the spectrum (Oppenheim et al., 1997). In such cases, the frequency spectrum is inaccurate. In our results, the apparent differences between the patterns of the Eff-TR2000 dataset and the TR400 dataset could be observed in all sub-bands between 0.1 and 0.25 Hz when we did not perform covariates regression. One plausible interpretation for this result is that if the nuisance covariates regression is not performed, then the high-frequency physiological noises could be aliased into the frequency bands that we actually observed (0–0.25 Hz) in the Eff-TR2000 dataset. Thus, the sensitivity and specificity in detecting the underlying differences are reduced. For the data with covariates regression, the effect of high-frequency noises was greatly reduced by regression; thus, the underlying differences were better revealed. Nevertheless, the discrepancy between the TR400 and the Eff-TR2000 datasets remained apparent in the sub-bands of 0.1–0.15 Hz. This effect might have occurred because the regressions of the WM, CSF and SC signals were insufficient for removing all potential physiological artifacts. fMRI experiments in which cardiac and respiratory signals are simultaneously recorded may be beneficial for revealing the effect of these physiological activities. Generally, our results supported that high sampling rate is superior to the conventional sampling rate for reliably capturing high-frequency AF differences. We recommend that high sampling rate should be used to study the changes in high-frequency rs-fMRI signals.

In summary, we observed significant differences in the amplitude of fast fluctuations of rs-fMRI signals between EO and EC resting states. The frequency bands in which the differences were detected were much higher than those studied conventionally. The results indicated that the high-frequency components of rs-fMRI signals can be modulated by different resting states; thus, considering the high-frequency information of rs-fMRI signals might prove beneficial in clinical studies. However, further work is needed to elucidate the functional significance of the fast fluctuations of rs-fMRI signals. Additionally, we reported substantial effects of nuisance covariates regression, and temporal sampling rate on the observed high-frequency results. Our results suggest that the confounding variables such as physiological signals and HM should be considered in future studies, and that a high sampling rate is necessary when investigating the high-frequency changes of rs-fMRI signals.

## Author contributions

Dong-Qiang Liu and Yu-Feng Zang conceived and designed the experiment. Bin-Ke Yuan and Dong-Qiang Liu performed the experiment. Bin-Ke Yuan and Dong-Qiang Liu analyzed the data. Bin-Ke Yuan, Jue Wang, Yu-Feng Zang and Dong-Qiang Liu wrote the paper.

## Conflict of interest statement

The authors declare that the research was conducted in the absence of any commercial or financial relationships that could be construed as a potential conflict of interest.

## Footnotes

^1^http://www.fil.ion.ucl.ac.uk/spm

^2^www.restfmri.net
